# Integrative taxonomic revision concerning phylogenetic relationships, morphological variation, distribution, ecology and conservation of *Gagea alberti* (Liliaceae) within its natural range

**DOI:** 10.1186/s40529-025-00465-z

**Published:** 2025-06-10

**Authors:** Serik Kubentayev, Igor Levichev, Marcin Nobis, Shukherdorj Baasanmunkh, Ewelina Klichowska, Aidyn Orazov, Daniyar Alibekov, Balsulu Kubentayeva, Hyeok Jae Choi

**Affiliations:** 1 Astana Botanical Garden, Astana, 010016 Kazakhstan; 2 Komarov Botanical Institute of RAS, Saint Petersburg, 190000 Russia; 3 Institute of Botany, Faculty of Biology, Jagiellonian University, Kraków, Poland; 4 Department of Biology and Chemistry, Changwon National University, Changwon, 51140 South Korea; 5 Laboratory of NatureLaB, Astana International University, Astana, 020000 Kazakhstan

**Keywords:** Biodiversity, Central Asia, *Gagea*, Goose onion, Phylogeny, Section

## Abstract

**Background:**

The genus *Gagea* is one of the largest and most complex members of the family Liliaceae, and comprises more than 320 species. *Gagea* is taxonomically challenging because of its morphological variations, polyploidization, hybridization and potential to inhabit ecologically diverse environments. In this study, we investigated the taxonomy, morphology, conservation, distribution and phylogeny, of *Gagea alberti* across within its natural range.

**Results:**

*Gagea alberti* iswidely distributed in Kazakhstan, Kyrgyzstan, southern Russia, western China, and Mongolia. This species exhibited high ecological plasticity and phenotypic variability. Based on extensive morphological and phylogenetic analyses, we treated *G. altaica* and *G. sarysuensis* as synonyms of *G. alberti*. The conservation status of this species was assessed as least concern (LC) at a global level. We provide detailed analytical and morphological illustrations of the ontogeny of *G. alberti*. Furthermore, we provide a checklist of section *Plecostigma*, including *G. alberti*, along with a brief morphological description of the section.

**Conclusions:**

This study provided new insights into the morphology, phylogeny, and distribution of *G. alberti*, a species characterized by high ecological plasticity and phenotypic variability. The high ecological plasticity and phenotypic variability of *G. alberti* are the results of a long-term evolutionary process that enabled the species to adapt to a wide range of environmental conditions.

## Background

The genus *Gagea* Salisb., distributed across Eurasia and along the Mediterranean coast of Africa, includes over 330 species (Levichev 2013a, b), making it the largest and most taxonomically complex genus in the Liliaceae family. The highest diversity of *Gagea* species is observed in the mountains of Western Tian Shan and Pamir-Alai, with 77 and 122 species, respectively (Levichev 1999a; Peterson et al. 2011; Kubentayev et al. 2023). In Kazakhstan, 63 *Gagea* species have been recorded (Kubentayev et al. 2021), with an uneven distribution across the country. The extensive plains of northern and central Kazakhstan host no more than 14 species (Kupriyanov 2020), whereas species diversity increases sharply in the western and southern mountains. Baitenov (2001) reported 12 endemic species of *Gagea* in Kazakhstan; however, the latest Kazakh endemic plant checklist (Kubentayev et al. 2024) includes only 4 species: *Gagea almaatensis* Levichev, A. Peterson & J. Peterson, *G. sarysuensis* Murz., *G. ularsaica* Levichev, and *G. iliensis* Popov.

*Gagea* is characterized by complex morphological features that combine both archaic and evolutionarily advanced traits (Peterson et al. 2008). In addition to the bipolar, colonial and tiered structure of the shoot of *Gagea* (and other bulbous annuals) has been detailed and illustrated previously (Levichev 2005, 2011a, 2011b, 2013a, 2013b; 2017; Krasovskaya, Levichev 2013a, b), representatives of this genus demonstrate pronounced morphological variability of vegetative and generative organs with species-specific manifestation at different stages of ontogenesis (Levichev 1990a, 1999a). The combination of these factors creates objective taxonomic difficulties, resulting in a large number of species within *Gagea,* and its nomenclature is overloaded with synonyms.

*Gagea alberti* Regel was described in 1880 based on specimens collected from Wusu Town (northwestern China, near the border with Kazakhstan) (Regel 1879). This taxon is native to Central Asia, including China (the northern region of Xinjiang), Kazakhstan, Kyrgyzstan, and Russia (Altai) (POWO 2025). Traditionally, *G. alberti* has been classified within the section *Plecostigma* (Turcz.) Pascher (Peterson et al. 2016). In general, this section is named for the style splitting either to the base or halfway along its length. It is a prominent trait in only two species: *Gagea chinensis* Y. Z. Zhao & L. Q. Zhao and *Gagea pauciflora* (Turcz. ex Trautv.) Ledeb. The three-lobed stigma is less distinctly expressed in other members of the section, as well as in nearly all representatives of the genus. The *Plecostigma* section is characterized by alternately arranged leaves on the flowering stem or at the nodes of a narrow, upward-elongated, few-flowered inflorescence, blunt or rounded tepals, an elongated, three-angled, prismatic capsule, and flat seeds (Levichev 1990a, b; Peterson et al. 2008).

*Gagea alberti* grows in various environmental conditions and exhibits considerable phenotypic variability, which complicates its identification. These difficulties arise due to the natural ontogenetic variability of shoot organs, small plant size, ephemeral development, short flowering period, and prolonged underground dormancy. Morphologically, *G. alberti* is most similar to *G. altaica* Schischk. & Sumnev and *G. sarysuensis* Murz. within the *Plecostigma* section. Recently, some studies have examined various characteristics of *G. alberti* and *G. altaica* in China, including leaf morphology, epidermal anatomy, and palynology (Lin et al. 2023; Qiu et al. 2023). However, its overall range, phylogenetic relationships, and biological features across diverse ecological conditions remain poorly understood.

The aim of this study was to conduct a comprehensive survey of *G. alberti*, including an analysis of its distribution, morphological characteristics, ecological and coenotic preferences, and phylogenetic and morphological relationships with closely related species, and to assess its current conservation status. The obtained data will provide a new perspective on the taxonomic position of *G. alberti*, clarify its intraspecific variability, and identify factors affecting its distribution and conservation.

## Materials and methods

### Plant material

Data on the distribution of *G. alberti* were obtained from collection sites documented for cultivation in the living collection of the genus *Gagea* (Levichev 2002), targeted field observations in Kazakhstan and beyond, the literature, herbarium collections (LE, AA, MW, NUR, KUZ, MHA, PE, TASH, and TK) (Thiers 2023), and observations on platforms such as iNaturalist (https://www.inaturalist.org/) and Plantarium (https://www.plantarium.ru/). Distribution maps were created using ArcMap software. A newly collected herbarium specimens were deposited at the NUR and LE.

Over 300 individuals have been analyzed in natural habitats and cultivated collections in Parkent, Tashkent and Saint Petersburg, including observations of seed germinants development (Levichev 1999b, 2002). Anatomical sections were prepared manually using a straight razor and, less frequently, a freezing microtome MZ-2. The examination was conducted under a microscope, with observations documented through hand drawings. Methodological approaches for creating analytical illustrations (species portraits) in accordance with Article 38.9 of the Shenzhen Code (Turland et al. 2018) and detailed diagrams of shoot structure at different stages of ontogeny have been previously discussed (Levichev 2013a).

### Phylogenetic relationship

The sequences of the internal transcribed spacer (ITS) for four individuals of *G. alberti* and one individual of *G. sarysuensis* were obtained from individuals we collected (a list of localities and individuals is presented in Appendix). The modified CTAB method used for DNA extraction and Sanger sequencing is described in Appendix. DNA sequences were deposited in the National Center for Biotechnology Information (NCBI) under accession numbers OR852564.1, OR852568.1, OR852566.1, OR852562.1, and OR852565.1. The remaining 41 representative sequences of the genus *Gagea* and 10 sequences from individuals belonging to closely related genera (as outgroups) were downloaded from the NCBI GenBank database (GenBank 2024) (accession numbers are included in Fig. 3).

The phylogenetic relationships of *Gagea* were inferred using the maximum likelihood phylogenetic trees constructed with MEGA v.11.0.13 (Tamura et al. 2021). The Tamura-Nei model with gamma-distributed rate variation across sites and a proportion of invariable sites, was chosen as the best-fit substitution model based on the Akaike information criterion (AIC) values. We used the bootstrap method with 1,000 replicati for the phylogenetic analysis.

### Species distribution modeling

The potential distribution model of *G. alberti* under current climate conditions was obtained using an ensemble modeling approach implemented in the R package ‘biomod2’ v.4.2.4 (Thuiller et al. 2023). We applied three commonly used models, i.e., maximum entropy (Maxent), generalized boosted model (GBM), and multivariate adaptive regression splines (MARS), to capture the variation in different ecological niche model (ENM) algorithms and enhance the robustness of the prediction (Araújo and New 2007; Araújo et al. 2019). We used 19 bioclimatic variables derived from WorldClim (Fick and Hijmans 2017) with a resolution of 0.05° (~ 5 km) as explanatory variables. We computed Pearson’s correlation for bioclimatic layers by using layerStats function in R package ‘raster’ (Hijmans et al. 2023) and removed autocorrelated variables (*r* > 0.7). For further modeling, we selected six variables (bio7, bio10, bio15, bio16, bio17, and bio19).

A total of 83 *G. alberti* locations were identified across four countries (Appendix): Kazakhstan (39 localities), Kyrgyzstan (1 locality), Russia (33 localities), China (5 localities), and Mongolia (2 localities). Among these, 34 locations were determined based on herbarium specimens, 21 were identified through observations on platforms such as iNaturalist (15 locations) and Plantarium (7 locations), and 25 were established through datasets available on the GBIF website. Due to the uneven distribution of points in different parts of the geographic range, localities closer than 10 km from each other were removed using the ‘thin_b’ (Heming et al. 2018) function. Finally, we used 54 occurrences of *G. alberti* for the distribution model. We applied ENMevaluate_b (ENMwizard) (Heming et al. 2018), testing various combinations of feature classes (linear, quadratic, product, threshold, hinge, and their hybrid combinations) and regularization multipliers (RM; from 0.5 to 5 with an increment of 0.5) (Heming et al. 2018) to optimize the model selection. We excluded distribution records with the 10% lowest probability detected using ENMeval (Muscarella et al. 2014), to enhance forecasting accuracy. Due to the lack of true absence data, we randomly generated 10,000 pseudo-absence points (background data) that present the available envirmental in the studied area, which is a commonly used method in ENM (Phillips et al. 2009; Thuiller et al. 2023). To do this, we used the BIOMOD FormatingData function with the ‘random’ method (Thuiller et al. 2023) and selected pseudo-absences from the area limited by a two-degree buffer width around the species occurrence polygon. We assigned equal weights to presence data and background points (prevalence of 0.5) (Barbet-Massin et al. 2012). Models were cross-validated with ten repeats by randomly splitting the distribution records into two datasets used for calibration (70%) and testing (30%). We removed the models with AUC < 0.8 or TSS < 0.6 from the final mean ensemble models to increase the prediction accuracy (Bellard et al. 2013). We used ArcMap10.5 (ESRI 2016) to visualize the results.

### Conservation assessment

The IUCN Red List criteria place taxa into one of six categories—critically endangered (CR), endangered (EN), vulnerable (VU), near threatened (NT), least concern (LC), or data deficient (DD)—based on current guidelines and criteria defined by the IUCN Standards and Petitions Committee (IUCN 2012). In particular, five different criteria have been defined by the IUCN (2012): population size reduction (criterion A); geographic range size fragmentation, few locations, range decline, or population fluctuations (B); small and declining population size and fragmentation, fluctuations, or few subpopulations (C); very small population or very restricted distribution (D); and quantitative analysis of extinction risk (E). In this study, the conservation status of *G. alberti* was assessed by the Geospatial Conservation Assessment Tool (GeoCAT; https://geocat.iucnredlist.org/; Bachman et al. 2011) based on 2 km^2^ grid cell size (IUCN 2012). This tool performs rapid geospatial analysis based on georeferenced data and automatically evaluates the conservation status solely based on the extent of occurrence (EOO) and area of occupancy (AOO) values (Bachman et al. 2011). The categories and criteria of threatened species were defined according to IUCN Standards and Petitions Committee (2024).

## Results

### Morphology and biology of *Gagea alberti*

**Fig. 1:**
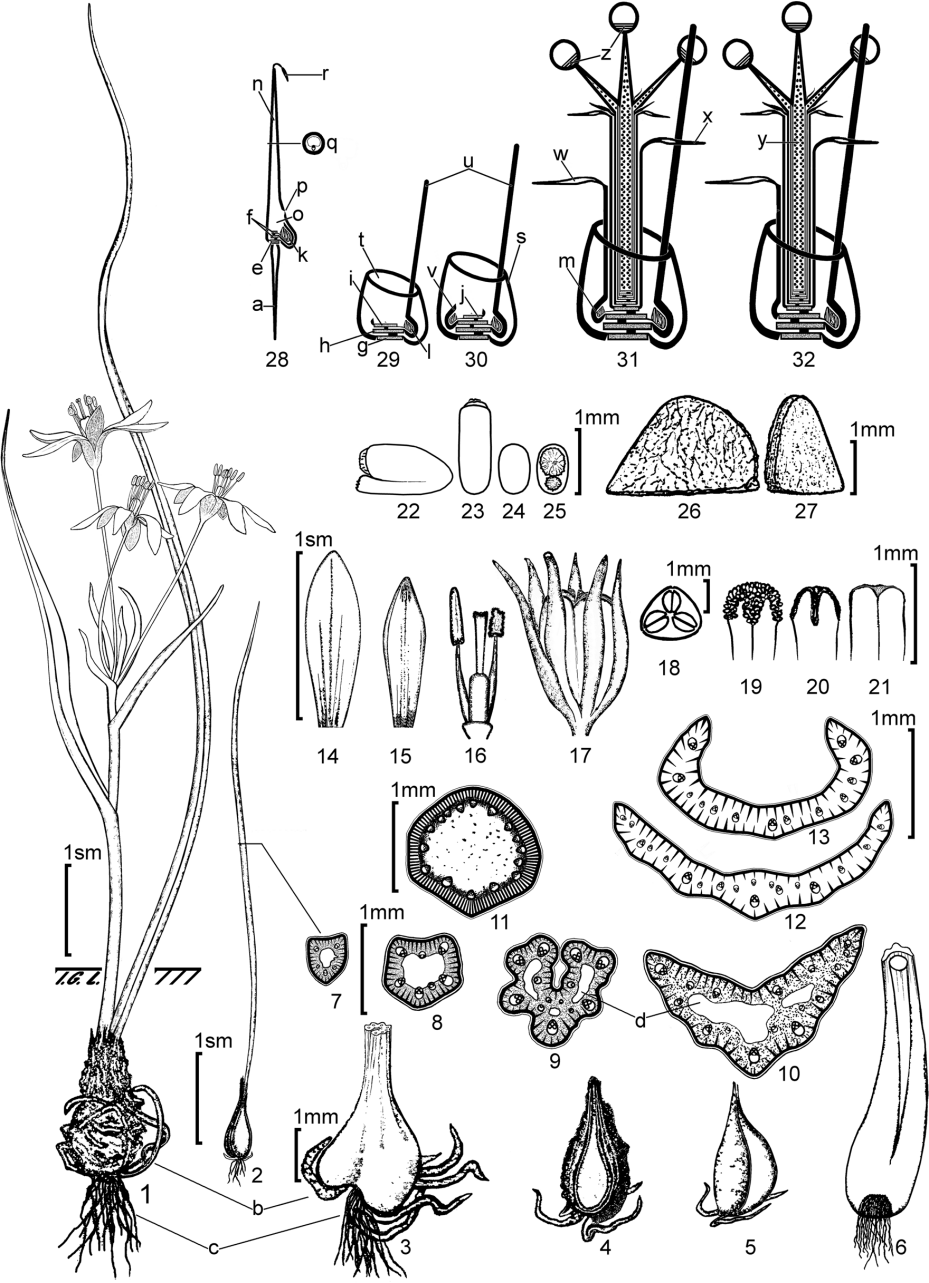
Analytical drawing and shoot structure of the main ontogenetic stages of *Gagea alberti*: 1. The general appearance of a generative individual. 2. One-year-old sprouts from the vegetative bulb. 3. Bulb of juvenile plant 2–4 years old with vegetative bulb, clarified (b) and thin (c) roots (dead sheaths removed). 4. Vegetative bulb from the side of attachment with its sclerified roots. 5. Vegetative bulb, turned by 1/3. 6. Bulb of an old generative individual without vegetative propagation (dead sheaths removed; see also 32). 7–10. Section of basal leaf: at 1 year (7), at 2nd year (8, see 29), at 3–4 years of life (9, see 30), at generative age (10, see 31). 11. Section of peduncle under the lower leaf. 12–13 Section of the lower leaf on the peduncle: in the first year of flowering (12), in the generative period (13, see 31w). 14–15. Tepals: outer (14), inner (15). 16. Ovary, style, filaments with anthers (opened on the right). 17. Mature capsule with dry tepals. 18. Section of ovary. 19–21 Stigma (entire): with papillae before pollination (19), after pollination (20), in a mature capsule (21). 22–25 Anatropous ovule: lateral view (22), dorsal view (23), view from the chalaza side (24), view from the side of attachment and micropyle (25). 26. Mature seed (side view). 27. Seed from the chalaza side and with a ¾ turn. 28–32. Schemes: seed sprout (28), 2nd year individual from seed, the same as in the 1st year from vegetative bulbil (29), 3–4 years of life (30), young generative individual (31), mature individual (32). *Letter designations:* a—primary root of seed sprout; b—thickened sclerified roots (underground velamen); c—thin feeding roots; d—cavity (fistula). Axial (stem) structures of the base (roots not shown): e—hypocotyl, n—forms a unifacial cotyledon, f—epicotyl, k—forms a covering reduced leaf around the primary replacement bulb. Axial structures of the bulb of a dicyclic shoot: g—is the axis of the storage ring scale (s, first cycle); structures of the second cycle: h—is the axis of the first basal leaf (u), i—is the axis of the second basal leaf (with its primordium), j—is the axis of the genuine sub inflorescence leaf (with its primordium). Bulbs: k—primary replacement bulb of seedling, l—replacement bulb of shoot n + 2, m—vegetative bulb. Leaf structures: n—unifacial cotyledon, o—cavity of cotyledon sheath, p—cotyledon outlet opening, q—cotyledon cross-section, r—haustorium, s—basal storage scale, t—outlet opening of basal scale, u—lamina of first basal leaf, v—rudiment of second basal leaf, w—free part of lamina of second basal leaf, x—free part of lamina of genuine sub inflorescence leaf, y—peduncle (in the form of phyllocladium); z—subflorescence disk (upper bottom)

**Fig. 2:**
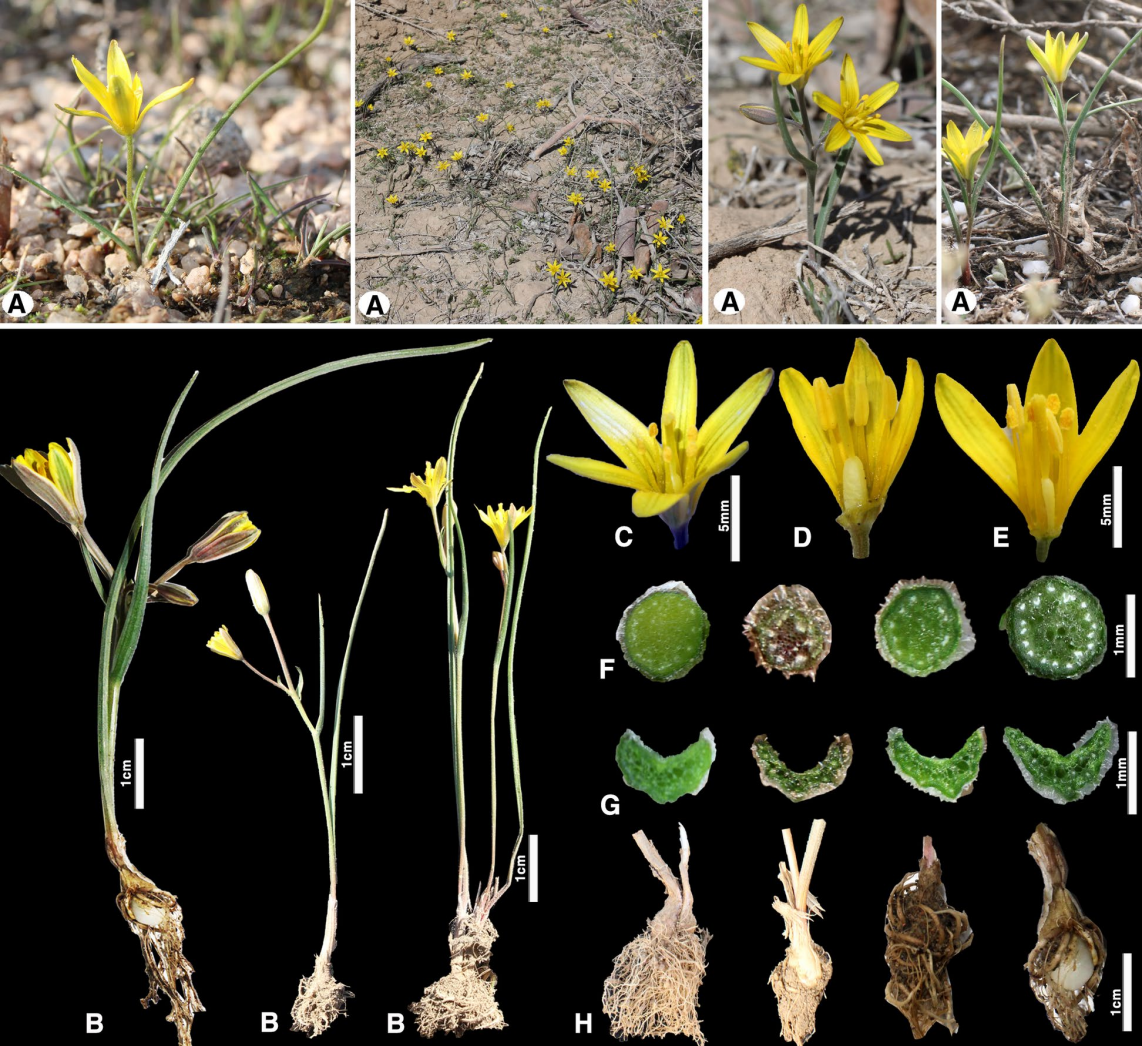
*Gagea alberti* in Kazakhstan: **A** general habitats; **B** habits **C**–**E** flowers tepals; **F** peduncle cross section; **G** transverse section of a basal leaf; **H** bulb with thickened sclerified and suckering roots. (Photos: A–H by S. Kubentayev)

*Gagea alberti* is a small bulbous ephemeral plant, 3–20 cm tall (depending on the ecological conditions), that grew either solitarily or in small groups of 2–8 individuals. The bulb (Fig. 1 (3)) was ovoid and enveloped in thickened sclerenchymatous roots in an ageotropic manner (Fig. 1 (3b), Fig. 2H) and had a geotropic bundle of thin feeding roots (Fig. 1 (3c)). It was covered with greyish-brown, tightly leathery, longitudinally split tunics that extended to the soil surface, forming a neck 1–7 cm long (Fig. 2B). Vegetative reproduction occurred during the juvenile period via a single, flat-convex bulblet (Fig. 1 (4, 5)) with a lower or, less commonly, central attachment. The peduncle, similar to the entire plant, was short and pubescent. The solitary basal leaf exceeded the inflorescence in length, was 1–3 mm wide, channel-shaped, and longitudinally ribbed. The peduncle had two alternating leaves, whereas the other leaves were clustered in a whorl at the base of the pedicels in the sparse inflorescence. The perianth segments (Fig. 1 (14, 15)) were 10–13 mm long, lanceolate, rounded, bright yellow inside and brownish-green, with whitish edges on the outside. The filaments (Fig. 1 (16)) were linear and pointed. The anthers (Fig. 1 (16)) were basifixed, oblong, and half their original lengths when dehisced. The style (Fig. 1 (16)) was linear and complete, with a shallow three-lobed stigma. The ovary (Fig. 1 (16, 18)) was oblong and prismatic. The ovules were anatropous. The mature seeds (Fig. 1 (26, 27)) were thin and flattened. The capsule (Fig. 1 (21)) was rounded and three-angled, slightly shorter than the perianth segments. A comprehensive analytical drawing of the shoot structure and morphology at the main stages of ontogenesis in this species is presented in Fig. 1.

We found that the peduncle functions as a type of phylloclade (Fig. 1 (32y)), integrating axial and foliar structures that develop congenitally (and thus fuse) within the bud and grow under a standard cover of leaf tissues above the basal hypopodia, terminating in the subfloral discs. Owing to the bipolar organization of their constituent metamers, each structural element of flowers in vascular plants (except embryos in ovules) contributed to shoot reinforcement through a system of vascular bundles that descend geotropically and emerge as roots in the soil. Embryos formed within the ovules of the ovary and surrounded by the endosperm represent a fundamentally new generation of individuals relative to the parent plant. Each embryo was oriented at 180° relative to the parent metamers. After completing morphogenesis under favorable conditions, each seed developed individually and autonomously, absorbing the endosperm through a haustorium at the upper end of the cotyledon (Fig. 1 (28r)). A single primary root (Fig. 1 (28a)) formed geotropically and emerged from the micropyle at the base of the hypocotyl (Fig. 1 (28e)). All subsequent shoot metamers were constructed sequentially and agotropically, based on the structural template of the embryo.

### Ecology of habitat and habitat-related morphological variability of *Gagea alberti*

*Gagea alberti* grows in various habitats, including the foothills of granite hills and rock crevices, semi-desert plains, hilly sands in deserts, slopes, and summits of dry, ancient, rocky, destroyed hills, and halophytic meadows. The ecology of growth was reflected in the morphological variability expressed in fluctuations in height above the ground, size of the collar of dead leaves, number of flowers in the inflorescence, and length and width of the leaves, tepals, flowers, and capsules while maintaining general structural features in the ontogenesis of the shoot and underground organs. A comparison of a large number of living (Fig. 2) and herbarium specimens of this kinship group in the territory of the Republic and from neighboring countries led to the conclusion that the taxon is characterized by natural variability, which led to the description of two names (*Gagea altaica* Schischk. & Sumnev. and *G. sarysuensis* Murz.).

### Phylogenetic analysis

**Fig. 3:**
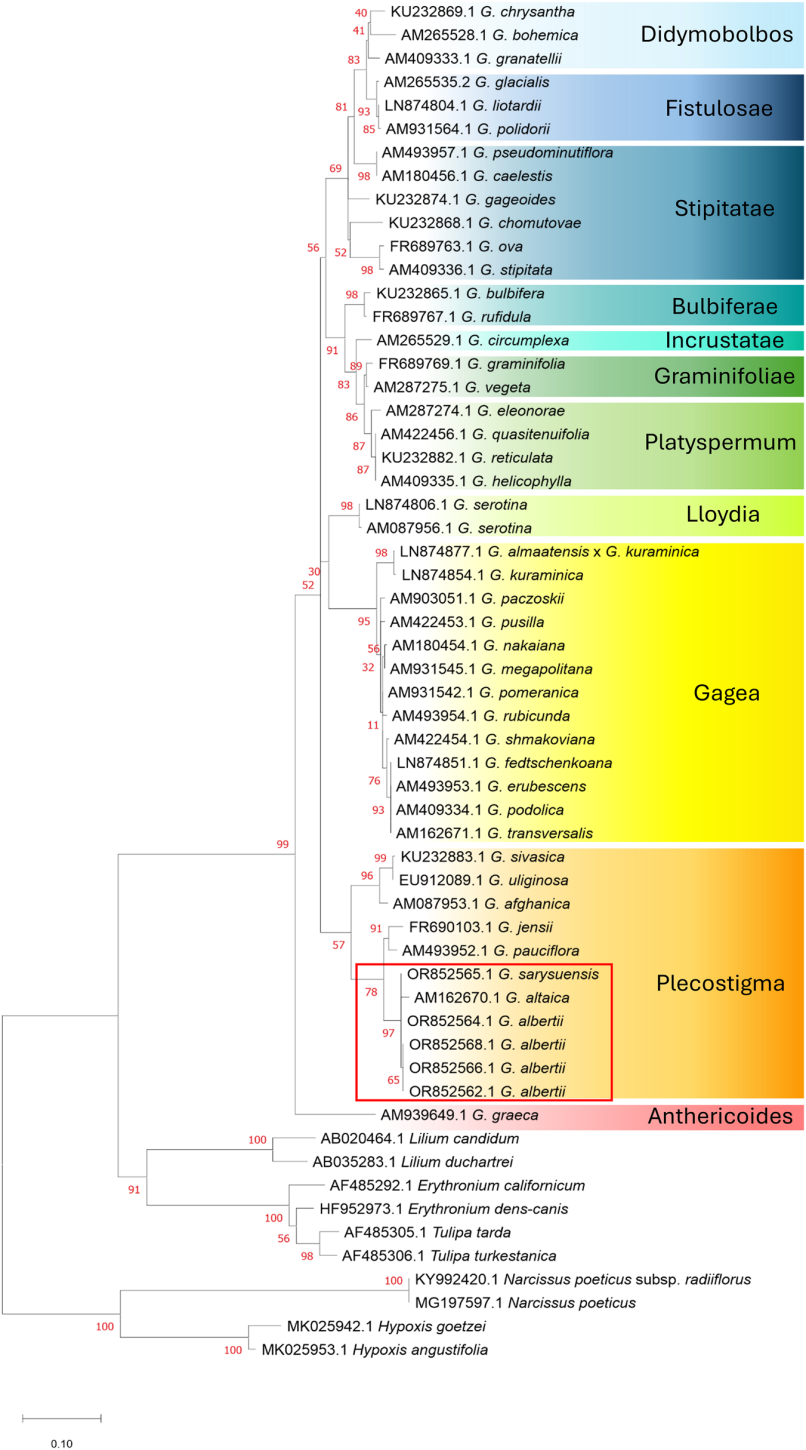
The Maximum Likelihood phylogenetic tree of the genus *Gagea* based on 496 bp of internal transcribed spacer (ITS) with bootstrap values (in red). The analysis involved 48 *Gagea* individuals and 10 individuals of closely related genera as an outgroup. The scale bar refers to substitutions per site phylogenetic distance

In accordance with the phylogenetic analysis based on ITS, *G. alberti*, *G. sarysuensis* and *G. altaica* were grouped in a polythomic subclade sister to that formed by *G. jensi* and *G. pauciflora* as well as to the clade of *G. sivasica*, *G. uliginosa* and *G. afghanica* representing the section *Plecostigma*. This group of species is grouped in a common sister clade to the one comprising all the rest of the analyzed *Gagea* taxa (Fig. 3). The ITS analysis supported the results of the morphological examinations, in which all three taxa, i.e., *G. alberti*, *G. sarysuensis*, and *G. altaica*, represented one conspecific species rather than three independent taxa.

### Current potential distribution of *Gagea alberti*

**Fig. 4:**
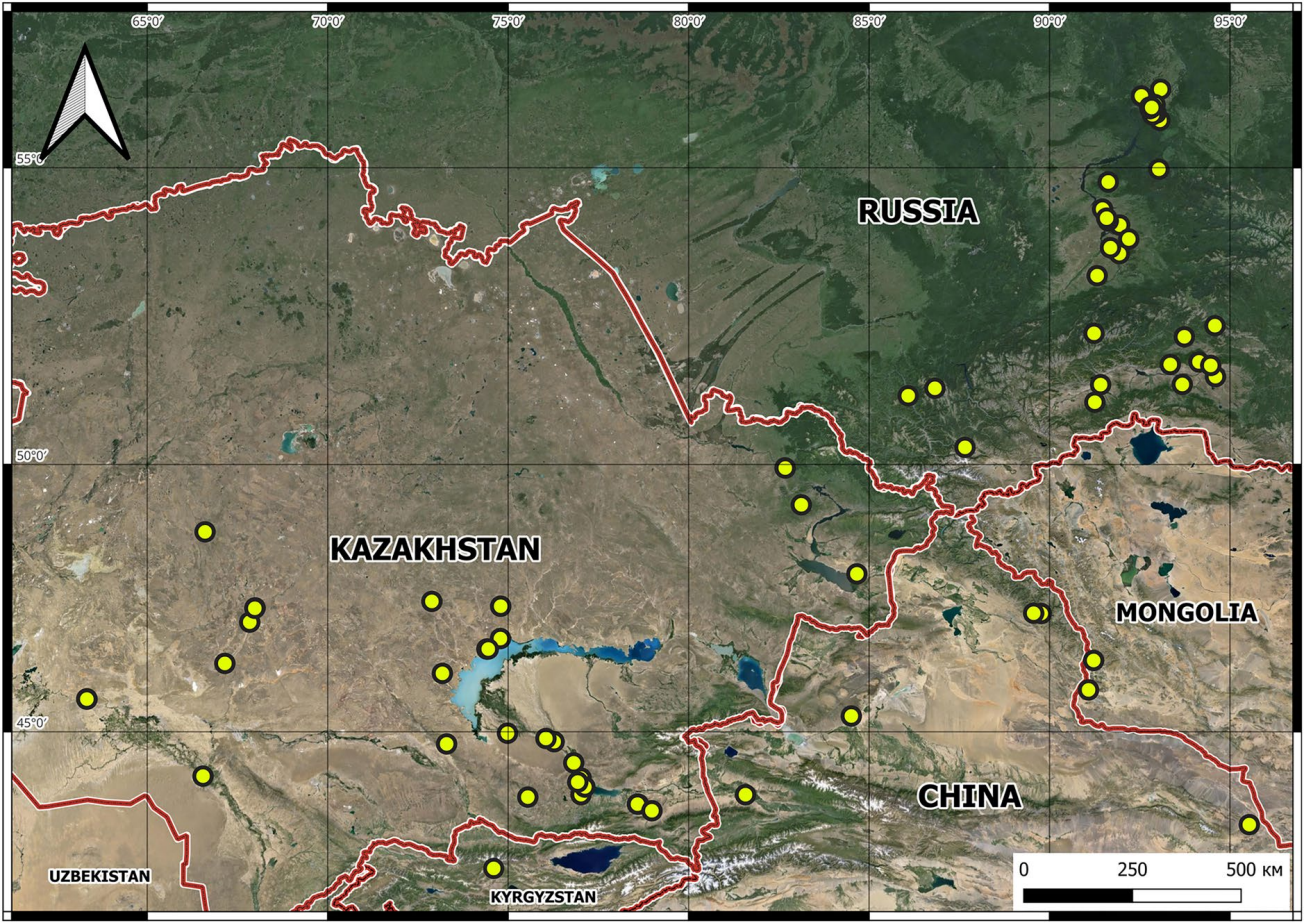
General distribution map of *Gagea alberti*

**Fig. 5:**
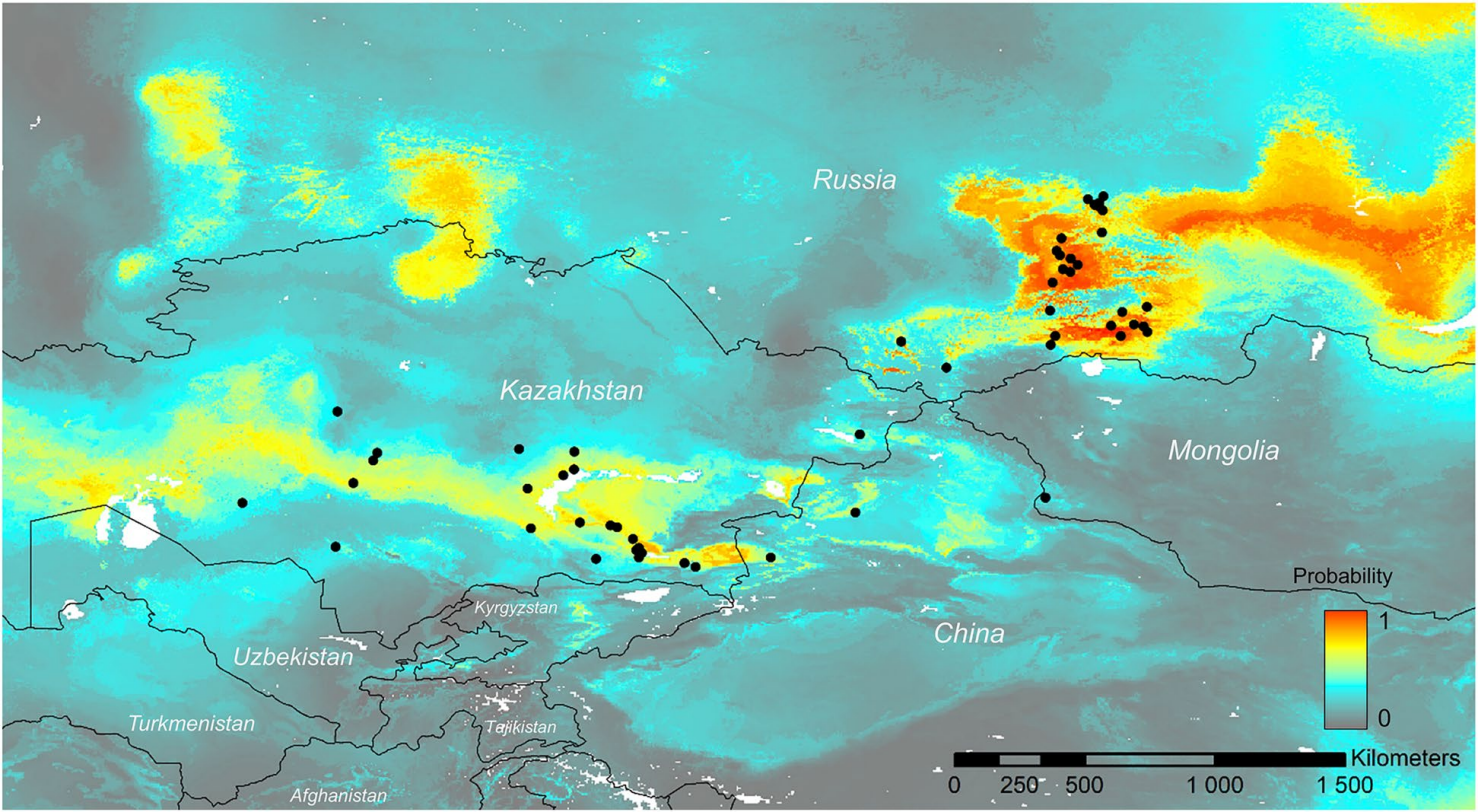
Model of the potential distribution of *Gagea alberti* in central Asia; probability of occurrence: very low (grey), medium (blue), high (yellow), very high (red)

The overall range of the species encompassed Central, Southern, and Eastern Kazakhstan, Kyrgyzstan, the Altai region in Russia, the northern part of the Xinjiang Uyghur Autonomous Region in China,and Western Mongolia (Fig. 4). The greatest number of localities of *G.alberti* is known from Kazakhstan (39 localities) and Russia (33 localities). The westernmost boundary of this species range reached the Ulytau region (Sarlyk village) in northern Kazakhstan and the Kyzylorda region (near Baikonur) in southern Kazakhstan. However, the model of the potential distribution of *G. alberti* indicated that the Altai and Sayan Mountains have the highest occurrence probability for this species (Fig. 5). Although this species occurs in the Altay Mountains, it has not been recorded in Sayans to date, and further research in this region is needed to confirm it as a potential hotspot for this species. It is also worth mentioning that the occurrence probability indicated by the model for this species was mostly medium, despite the numerous localities of *G. alberti* in Kazakhstan. Of the six variables used in the potential distribution model analysis, three (Bio15: precipitation seasonality; Bio10: mean temperature of the warmest quarter; and Bio19: precipitation of the coldest quarter) had the greatest impact on the model (Table 1).

**Table 1:** The importance values of bioclimatic variables involved within the model

Bioclimatic variables	Abbreviation	Variables importance [%]
Precipitation seasonality (coefficient of variation)	bio15	48.6
Mean temperature of warmest quarter	bio10	36.5
Precipitation of coldest quarter	bio19	18.4
Precipitation of driest quarter	bio17	16.8
Temperature annual range	bio7	11.2
Precipitation of wettest quarter	bio16	9.3

## Discussion

The representatives of the section *Plecostigma* originated in the microthermal environment of high-altitude regions, enabling their dispersal across the mountains of Central Asia and, in some cases, reaching Yakutsk (*G. provisa* Pascher). Significant geological events such as landslides, mudflows, and erosion in mountainous systems play a role in transporting bulbs, bulblets, and seeds with altered genomes to lower elevations. These processes, coupled with the ongoing mountain uplift, have facilitated new hybrid interactions and the movement of plants to higher altitudinal zones (Levichev 2013a, b). Molecular phylogenetic studies of the genus *Gagea* consistently demonstrated that members of the section *Plecostigma* belong to one of the tightly branching clades in phylogenetic trees (Peterson et al. 2008, 2009, 2011, 2016, 2019; Peruzzi 2008; Zarrei et al. 2009, 2010, 2011, 2012), which our data confirmed this distinction (Fig. 3).

No mechanisms underlying bulb burial during ontogenesis have been identified within the section *Plecostigma*. Observations of *G. alberti* indicate that the neck length (formed from dead leaves and peduncle remnants) typically varies within a single population. The neck usually reaches the soil level and rarely extends beyond it. Their dimensions may depend on the origin of the individual plants. Seed-propagated specimens with surface-level germination exhibited short necks. Simultaneously, older individuals buried by wind or water deposition in fine soil can regenerate vegetatively over generations at the depths of the parent plant. Consequently, groups of such individuals accumulate necks of uniform length, as observed in the type specimens. This phenomenon likely applies to the other 18 species in this section that exhibit varying degrees of neck elongation.

Not all representatives of the *Plecostigma* section exhibit the development of thickened sclerified roots (Fig. 1b). Developing as biennials (Levichev 2001), species of the genus *Gagea* produce two types of annual roots. Sometimes, the difference in their thickness is not noticeable. Typically, in older plants, thickened roots do not form at all, or short thickened roots are characteristic only of vegetative bulbs, as seen in *G. alii, G. chinensis*, and *G. vvedenskyi*. Thus, clarified roots are a distinct species-specific feature only in *G. alberti, G. damascena, G. deserticola,* and G*. korshinskyi*. In other species, they are virtually indistinguishable from thin feeding roots.

Challenges in the species identification of *Gagea* are primarily due to its vast geographical range and insufficient field collections for detailed comparisons. Recognized patterns of morphological variability during ontogenesis, polyploidization, and hybrid interactions are well documented (Levichev 1990b, 1999b, 2011a, b; Peterson et al. 2008, 2011b, 2016; Peruzzi 2008; Peruzzi et al. 2011). This relatively young and actively diverging genus exhibits high natural variability in morphological traits (Levichev 2013a; Wörz et al. 2012; Peterson et al. 2016). The basal organs of the shoots are remarkably diverse. In contrast, the upper parts, especially the flowers and their components, exhibited a high degree of uniformity and similarity, reflecting their earlier evolutionary emergence than the bulbs. Consequently, basal organ characteristics are considered more reliable taxonomic markers than floral traits (Levichev 1999b, 2011b).

Our study on the morphology and variability of *G. alberti* strongly supported this conclusion. Despite differences in plant and organ size, characteristics such as cross-sections of basal leaves, bulb structure, and vegetative reproduction serve as reliable taxonomic markers for species delimitation within the genus *Gagea* (Levichev 2011b). In contrast, morphological similarities supported by phylogenetic analysis confirmed that *G. altaica* and *G. sarysuensis* should be treated as synonyms *G. alberti*. Phylogenetic studies are based on constructing a tree using the ITS region, which has demonstrated high effectiveness in the taxonomic studies of *Gagea*. (Peterson et al. 2008, 2011, 2016). Variations in the size of *G. alberti* plants and their organs may be attributed to environmental and habitat conditions. Qiu et al. (2023) noted a high morphological similarity between *G. altaica* and *G. alberti*, and located them as closely related. Additionally, Lin et al. (2023) also noted a close relationship between *G. altaica* and *G. alberti* based on a study of pollen morphology*,* referring them to the same section. However, they found some differences in the ornamentation of the exine of these species.

Numerous terminological discrepancies regarding the peduncle have been previously discussed (Levichev 2013a). The study of *G. alberti* morphology has enabled the characterization of the axial and foliar structures in bulbous plants as a phylloclade. In bulbous plants, each metamer forms a single descending vascular bundle (a structural element or root), penetrating the preceding metamers and uniting with other root-like structures. These findings are consistent with those of previous studies (Tomlinson 1995). In this integrated state, the vascular bundle structure emerges as an adventitious root above the primary root collar (Levichev 1999a, 2013a; Levichev et al. 2011).

Reports on *G. alberti* in Kazakhstan was previously limited to Kazakh Altai and Tarbagatai (East Kazakhstan region) under the names *G. altaica* (Goloskokov 1958; Kotukhov 2005), the Kazakh Upland (Karaganda region) (Kupriyanov 2020), and the Dzungarian Alatau (Zhetysu region) (Goloskokov 1958). This study significantly expanded the known range of this species, including new records from Mongolia and the westernmost limits in the Ulytau and Kyzylorda regions of Kazakhstan.

### Taxonomic treatment

***Gagea alberti*** Regel.

**Fig. 6:**
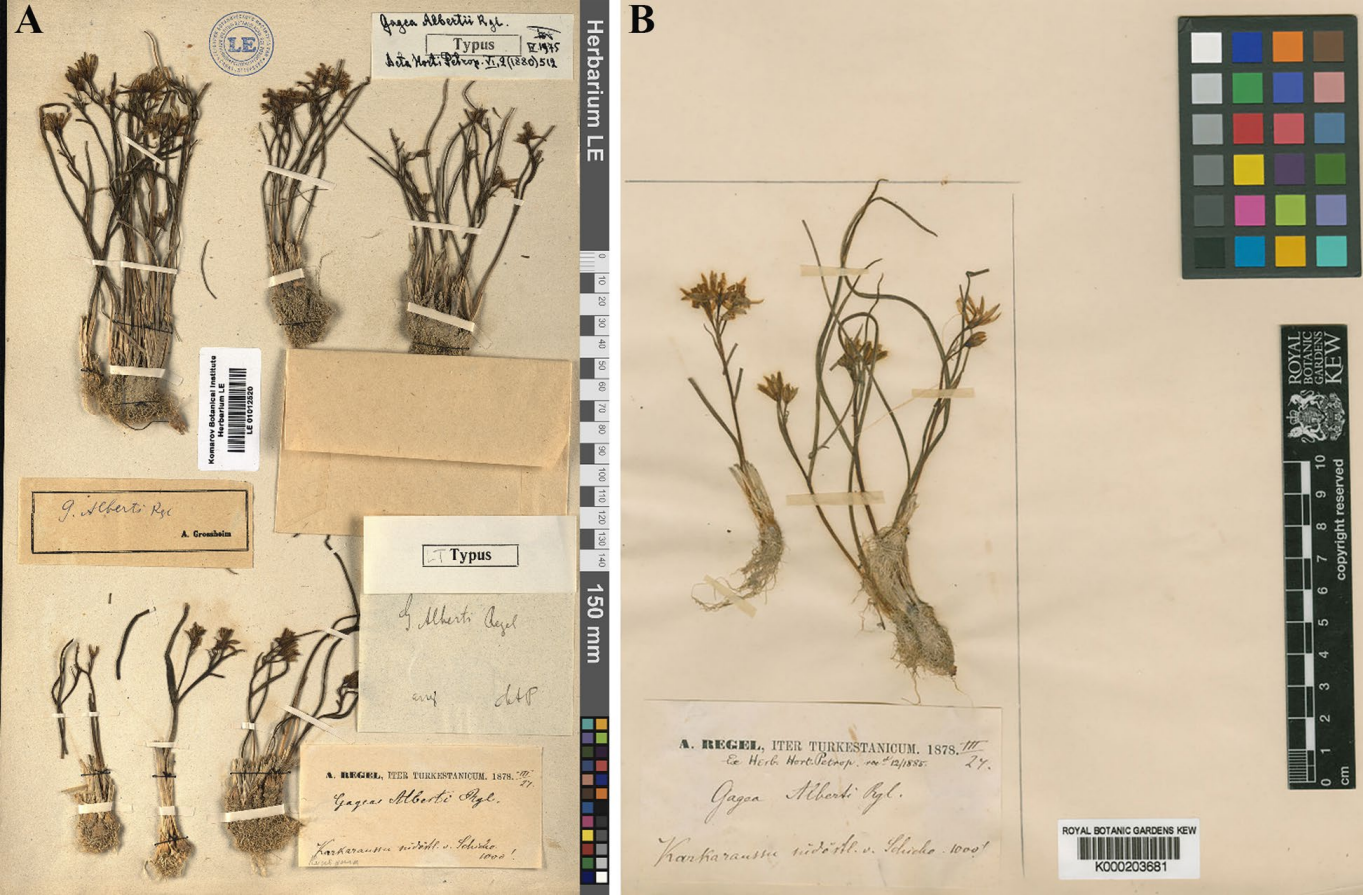
The type specimens of *Gagea alberti*. **A** holotype (LE01012520!); **B** paratype (K000203681) with original labels by E. Regel and autographs by A. Pascher (≈1907), A. Grossheim (1932), and V. I. Grubov (1975)

Holotype: China «Karkara ussu non procul a Schicho, 1000’ (24.03.1878. A. Regel» LE01012520! Isotype: K000203681!, P!), (Fig. 6A, B).

 = ***Gagea altaica*** Schischk. & Sumnev. syn. nov. Lectotype: Kazakhstan, «Eastern Kazakhstan as being in the "vicinity of Ust-Kamenogorsk city, rocky mountain peaks, 11.04.1926, *G. Sumnevich*» designated by: AV Polozhij, VF Balashova. The type specimen is stored in the Herbarium of Tomsk State University (TK).

 = ***Gagea sarysuensis*** Murz. syn. nov. Type: Kazakhstan, «Sarysu River (a tributary of the Kengir River), gravelly-fine earth coastal areas, 20.04.1990, *G.Zh. Murzalieva».* The type specimen is stored in the Herbarium of the Institute of Botany and Phytointroduction (AA!).

**Fig. 7:**
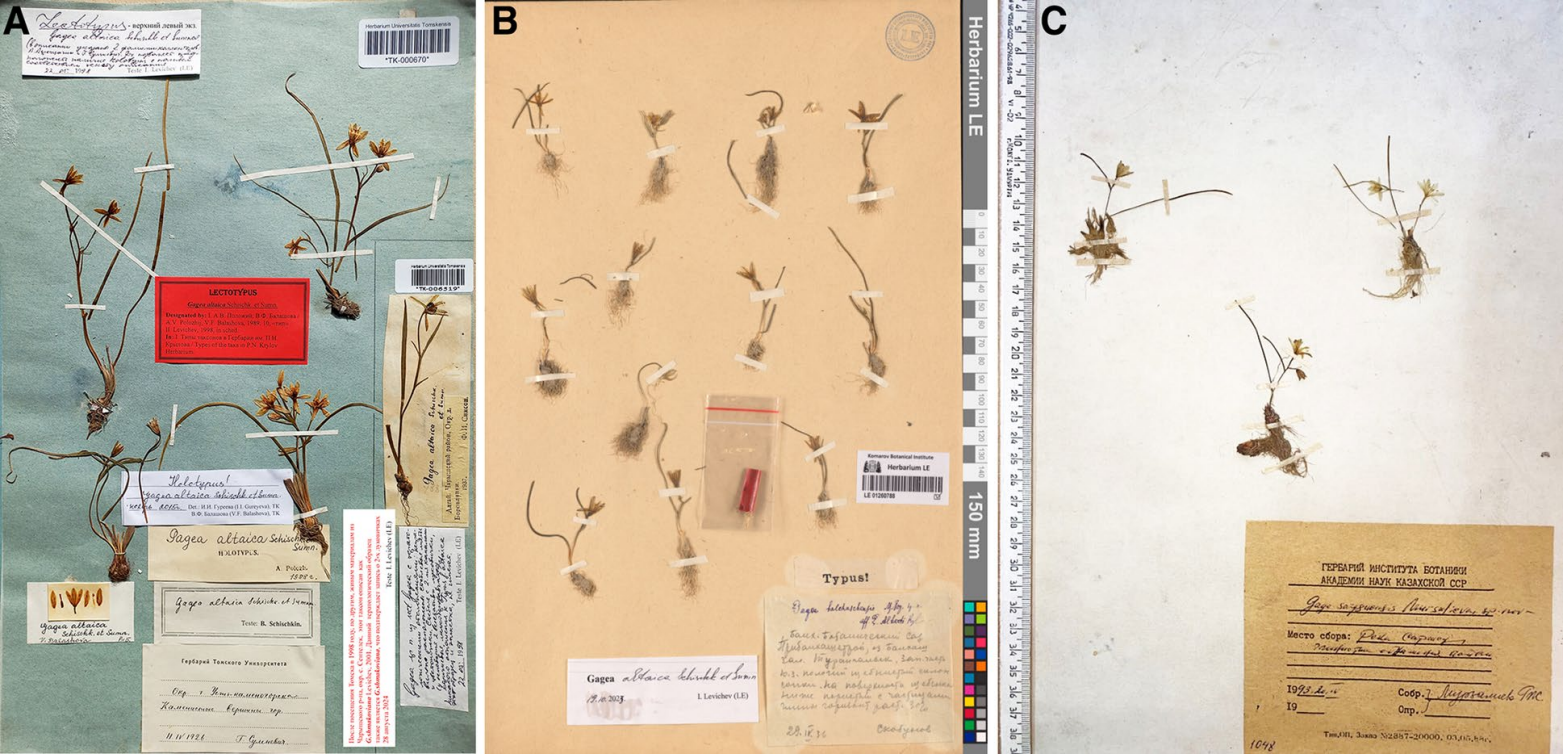
Type specimens of *Gagea* species. **A** Lectotype of *G. altaica* (TK000670!); **B** Holotype of *G. balchaschensis* (LE01260788!); **C** Holotype of *G. sarysuensis* (AA!)

*Gagea altaica* was first described by Schischkin & Sumnevicz in 1928. It was found on rocky mountain peaks in Eastern Kazakhstan. However, we did not find any significant differences between *G. altaica* and *G. alberti* based on their type specimens or wild populations (Figs. 1, 2, 6, 7A). In addition, the irregular occurrence of an additional leaf in the axil of the lower sub-inflorescence leaf was observed in both herbarium specimens. This characteristic was illustrated in Flora Reipublicae Popularis Sinicae (1980). Similar features have been observed in many natural populations. Therefore, we suggested *G. altaica* as an additional synonym for *G. alberti*.

We have been searching for *G. sarysuensis* (Fig. 7C) in its type locality in the Sarysu River (a tributary of the Kengir River), which is a gravelly, fine-grained coastal area, over the past four years. However, we did not find any representative species related to *G. sarysuensis*, except for various forms of *G. alberti*. These included tall plants (8–12 cm in height) growing on loamy soils in communities dominated by *Artemisia terrae-albae* Krasch and small plants (3–5 cm in height) growing on the gravelly banks of the Sarysu River. By comparing the type specimen of *G. sarysuensis* with collections of *G. alberti* from the Sarysu and Kengir Rivers, we concluded that the type specimen of *G. sarysuensis* falls within the range of morphological variability of *G. alberti*. The description and other elements of the protologue of *G. sarysuensis* fully aligned with the low-growing specimens of *G. alberti* collected from the Sarysu River valley. Kupriyanov (2020) reported *G. sarysuensis* in Bektauata (Karaganda Region) based on herbarium material: "Bektauata Mountain near the Karabuta wintering site, 18.04.2009, Kupriyanov A.N., Manakov Yu.A. (KUZ!)". Upon examining this specimen and collecting fresh material from the site, we determined that all collections from Bektauata belonged to *G. alberti*. Thus, it has been established that *G. sarysuensis* is morphologically indistinguishable from *G. alberti*, and this name should be treated as a synonym of the latter.

M.G. Popov selected specimens under the name *Gagea balchaschensis* ined. from the northern Balkhash region for the description (herbarium specimen is preserved in LE). Careful examination of these specimens (Fig. 7B) showed that the plants represented a miniature form of *G. alberti*, adapted to arid and harsh ecological conditions rather than separate taxa, because none of the key morphological characters, except for the height of the plant, differentiated them from each other.

#### Distribution and habitat

*G. alberti* is distributed throughout China, Kazakhstan, Kyrgyzstan, Mongolia, and Russia (Fig. 4). Based on our findings, this species is reported for the first time in Mongolia and the Ulytau and Kyzylorda regions of Kazakhstan.

#### Conservation status

The estimated extent of occurrence was 2,237,535 km^2^ and the estimated area of occupancy was 260 km^2^. Thus, *Gagea alberti* has been assessed as least concern at global level.

#### New findings

Mongolia: Dzungarian Gobi, Khovd Province, Uvkhod-Ula, 45° 49' N 91° 05' E, southern macroslope, among the rocks, *S.V. Smirnov *et al*.*, 05/06/2002 (LE); Khovd Province, Bayangol River valley, Arshantyn-Nuru ridge, near Bayan-Hutel pass, 46° 23' N 91° 12' E*, S.V. Smirnov *et al*.*, 05/12/2002 (LE). Kazakhstan: Kyzylorda region, Shieli district, near the town of Shieli, 44.126366N; 66.545515E, 04/20/2021, *S.A. Kubentayev* (NUR); Ulytau region, Ulytau district near the village of Sarlyk, 48.774823 N; 66.645094 E, 04/03/2024, *S.A. Kubentayev* (NUR).

#### A checklist of Gagea section Plecostigma

Section *Plecostigma* (Turcz.) Pascher, 1904. Lotos, 52, 24: 116; id., 1907, Bull. Soc. Nat. Moscou, 19: 374.

TYPE: *Plecostigma pauciflorum* Turcz. = *Gagea pauciflora* (Turcz. ex Trautv.) Turcz. ex Ledeb.).

The section *Plecostigma* includes 20 taxa which are characterised by alternately arranged leaves on the flowering stem or at the nodes of a narrow, upward-elongated, few-flowered inflorescence. The tepals are blunt or rounded. The capsule is elongated, three-angled, and prismatic. The seeds are flat (Levichev 1990a, b). All axial structures at the base of the shoot (including the nodes) are concentrated in the basal plate, which serves as a kind of stem in bulbous plants. The axial structures above the flowering discs, accordingly, can be referred to as apical-basal plates (Levichev 2013a). The flowering stem is rounded in cross-section, with a weakly expressed abaxial rib (a result of fusion with the basal leaf). The basal leaf is solitary, except in *G. pauciflora*, where both basal leaves are free during the first year of flowering. Subsequently, the second basal leaf fuses with the flowering stem and separates from it slightly below the inflorescence, forming a sub-inflorescence leaf. The remaining leaves (bracts) are usually clustered at the base of the closely spaced pedicels in the whorl of a few-flowered inflorescence or are alternately arranged in single-flowered species. Most of the section is distinguished by single-flowered species (rarely with two flowers): *G. alashanica*, *G. chinensis*, *G. daqingshanensis*, *G. jaeschkei*, *G. jensii*, *G. kuraiensis*, *G. neo-popovii*, *G. soleimani*, *G. uliginosa*. After flowering and before capsule maturation, pedicels typically elongate by 1.5–2 times. Pedicels elongate significantly in *G. pauciflora*, up to three times their original length.

1. *Gagea alashanica* Y. Z. Zhao & L. Q. Zhao, Acta Phytotax. Sin. 41(4): 393 (2003).
2. *G. alberti* Regel, Trudy Imp. S.-Peterburgsk. Bot. Sada 6: 512 (1879).
3. *G. alii* Levichev, Pakistan J. Bot. 38(1): 49 (50) (2006).
4. *G. chinensis* Y.Z.Zhao & L.Q.Zhao, Ann. Bot. Fenn. 41(4): 297 (2004).
5. *G. damascena* Boiss. & Gaill., Diagn. Pl. Orient. ser. 2, 4: 105 (1859).
6. *G. daqingshanensis* L. Q. Zhao & Jie Yang, Ann. Bot. Fenn. 43(3): 223 (2006).
7. *G. deserticola* Levichev, Turczaninowia 4(1–2): 9 (2001) (2001).
8. *G. goljakovii* Levichev, Turczaninowia 4(1–2): 12 (2001).
9. *G. jaeschkei* Pascher, Sitzungsber. Deutsch. Naturwiss.-Med. Vereins Böhmen 'Lotos' Prag. 1904: 128 (1904).
10. *G. jensii* Levichev & Schnittler, Organisms Diversity Evol. 11(5): 393 (-394) (2011).
11. *G. korshinskyi* Grossh., Fl. URSS 4: 77, 735 (1935).
12. *G. kuraiensis* Levichev, Turczaninowia 4(1–2): 16 (2001).
13. *G. neopopovii* Golosk., Bot. Mater. Gerb. Bot. Inst. Bot. Acad. Nauk Kazakhsk. S.S.R. 9: 8 (1975).
14. *G. pauciflora* Turcz., Bull. Soc. Imp. Naturalistes Moscou 27(2): 113 (1854).
15. *G. siphonantha* Rech.f., Pl. Syst. Evol. 153(3–4): 289 (1986).
16. *G. soleimani* (Bornm. ex Pascher) Rech.f., Fl. Iranica [Rechinger] 165: 43 (1990).
17. *G. uliginosa* Siehe & Pascher, Sitzungsber. Deutsch. Naturwiss.-Med. Vereins Böhmen 'Lotos' Prag. 1904: 127 (1904).
18. *G. utriculosa* Levichev, Pakistan J. Bot. 38(1): 52 (-53) (2006).
19. *G. vvedenskyi* Grossh., Fl. URSS 4: 107, 737 (1935).
20. *G. wendelboi* Rech.f., Pl. Syst. Evol. 153(3–4): 291 (1986).

## Conclusions

This study provides new insights into the morphology and distribution of *G. alberti*, a species characterized by high ecological plasticity and phenotypic variability. Two names, *G. altaica* and *G. sarysuensis*, have been identified as ecological forms of *G. alberti* and are now treated as additional synonyms for this species. The overall range of *G. alberti* covers central, southern, and eastern Kazakhstan, Kyrgyzstan,the Altai region in Russia, the northern part of the Xinjiang Uyghur Autonomous region in China, and the western parts of Mongolia (Fig. 4). The westernmost boundary of this species is the Ulytau region of Kazakhstan. The high ecological plasticity and phenotypic variability of *G. alberti* resulted from its long-term evolution. The data obtained can serve as a basis for further research on the taxonomy, morphology, and ecology of closely related species of the genus *Gagea*.

## Data Availability

Not applicable.

## Appendix: Method for DNA isolation

Dried leaves of *Gagea alberti* collected in Central and Southern Kazakhstan were used as research objects (Table 2). DNA sequences of *Gagea alberti* have been deposited in the National Center for Biotechnology Information (NCBI; Table 2).

**Table 2:** Data on Gagea alberti samples collected for molecular studies

Species name	Habitat	Coordinates	No. in NCBI
*Gagea alberti*	Kazakhstan, Lake Balkhash, Turangylyk Bay	46.600650N; 74.448827E	OR852564.1
*Gagea alberti*	Kazakhstan, Kyzylorda Region, Shieli District, near the town of Shieli	44.126366N; 66.545515E	OR852568.1
*Gagea alberti*	Kazakhstan, Karaganda Region, Targyl Mountain	46.701740N, 74.313127E	OR852566.1
*Gagea alberti*	Kazakhstan, Ulytau Region, Ulytau District, near the village of Sarlyk	48.774823 N; 66.645094 E	OR852562.1
*Gagea sarysuensis*	Kazakhstan, Sarysu River (a tributary of the Kengir River)	47.372929N; 67.984344E	OR852565.1

A modified CTAB method was used for DNA extraction. Leaf and herbarium samples were ground in a mortar until homogeneous in 1 mL of acidic CTAB buffer (2% CTAB, 2 M NaCl, 10 mM Na₃EDTA, 50 mM HEPES, pH 5.3), and the homogenate was transferred to 2 mL Eppendorf Safe-Lock tubes. Then, 800 µL of a chloroform-isopropanol mixture was added to the samples, vortexed intensively for one minute, and incubated in a thermal block at 60°C. The mixture was then centrifuged at 14,000 rpm for 5 min. The upper aqueous phase was carefully transferred to new tubes without disturbing the interphase. An equal volume of isopropanol (900 µL) was added to the supernatant, vortexed, and DNA was precipitated by centrifugation at 14,000 rpm for 10 min. The supernatant was discarded, and the pellet was washed with 1 mL of 70% ethanol and centrifuged again. The pellet was then dissolved in 200 µL of 1 × TE buffer (1 mM EDTA, 10 mM Tris-HCl, pH 8.0).

The DNA concentration was determined using a spectrophotometric method with a NanoDrop 1000 spectrophotometer (Thermo Scientific). Visualization of the extracted DNA was performed in a 1% agarose gel using a PharosFX Plus gel documentation system (Bio-Rad Laboratories Inc., USA). Electrophoresis was conducted at a constant voltage of 90 V for 120 min. Sequencing of the amplified DNA was performed using the ABI3700 capillary sequencer (Applied Biosystems, Thermo Fisher Scientific) with the Sanger method (BigDye® Terminator chemistry). PCR product purification was carried out using the ExoSAP-IT™ PCR Product Cleanup Reagent (Thermo Fisher Scientific) following the manufacturer's protocol. Multiple alignment of nucleotide sequences was conducted using Clustal Omega (https://www.ebi.ac.uk/Tools/msa/clustalo/).
